# State-transition trajectories in out-of-hospital cardiac arrest: neurological outcomes beyond myocardial activity

**DOI:** 10.1186/s13054-025-05815-8

**Published:** 2025-12-24

**Authors:** Kenji Kandori, Tasuku Matsuyama, Tetsuhisa Kitamura, Hiromichi Narumiya, Wataru Ishii, Masahito Hitosugi, Yohei Okada

**Affiliations:** 1 Department of Emergency and Critical Care Medicine, Japanese Red Cross Society, Kyoto Daini Hospital, 355-5 Haruobi-cho Kamigyo-ku, Kyoto, 602- 8026 Japan; 2 Department of Legal Medicine, Shiga University of Medical Science, Tsukinowa, Seta, Otsu, 520-2192 Shiga Japan; 3 Department of Emergency Medicine, Kyoto Prefectural University of Medicine, Kaji-cho 465, Kamigyo-ku, Kyoto, 602-8566 Japan; 4 Division of Environmental Medicine and Population Sciences, Department of Social and Environmental Medicine, Graduate School of Medicine, Osaka University, 2-2, Yamada-oka, Suita, 565-0871 Osaka Japan; 5 Department of Preventive Services, Graduate school of medicine, Kyoto University, Yoshida-Konoe-cho, Sakyo-ku, Kyoto, 606-8501 Japan; 6 Health Services and Systems Research, Duke-NUS Medical School, National University of Singapore, 8 College Road, Singapore, 169857 Singapore

We thank Rajsic et al. for their comments regarding the heterogeneity of pulseless electrical activity (PEA) and the role of point-of-care ultrasound (POCUS) in out-of-hospital cardiac arrest (OHCA) [1]. Although the distinction between “pseudo-PEA” and “true PEA” is theoretically appealing, these constructs remain unestablished in routine clinical practice and are not captured in major OHCA registries. Therefore, our analysis prioritized validated, standardized registry variables to maximize robustness and generalizability to real-world practice.

With respect to heterogeneity within non-shockable rhythm, we explicitly analyzed PEA and asystole as separate categories in our multivariable model. As shown in Fig. 1 and Supplemental Table 3 [2], the adjusted odds ratios (aORs) for favorable neurological outcome compared with shockable rhythm at hospital arrival were 0.23 (95% CI 0.13–0.40) for PEA and 0.07 (95% CI 0.03–0.17) for asystole, supporting our approach of treating them as distinct entities. Standard rhythm classification therefore captured clinically meaningful differences in outcomes within this large dataset.

Concerning the clinical utility of POCUS, the study cited by the authors (Teran et al.) suggests an association between myocardial activity and return of spontaneous circulation (ROSC) but does not demonstrate a statistically significant association with survival to hospital admission (OR 1.02, 95% CI 0.96–1.08) [3]. Because myocardial activity on POCUS does not reliably predict even hospital admission, its relevance to our primary and more rigorous endpoint of favorable neurological outcome remains unestablished.

Important differences in study design and patient populations further limit direct comparison. Prior studies using POCUS typically assessed myocardial activity at a single timepoint in patients presenting with PEA [3, 4]. In contrast, our registry-based analysis uniquely evaluates the dynamic trajectory from initial shockable rhythm to hospital-arrival status (ROSC, shockable rhythm, PEA, or asystole) [2]. Patients with initial shockable rhythm at the scene often have ischemic etiologies and may be considered for aggressive interventions such as extracorporeal cardiopulmonary resuscitation (ECPR), a context not addressed in the cited echocardiographic studies.

Finally, we reiterate that the ultimate aim of resuscitation is favorable neurological outcome, rather than ROSC or survival alone. Although pseudo-PEA indicates preserved mechanical myocardial activity, it does not necessarily ensure cerebral viability. Conversion from initial shockable rhythm to PEA may reflect progression toward the “metabolic phase” of cardiac arrest and irreversible ischemia–reperfusion injury [5]. Reliance on myocardial activity on POCUS, which is associated with ROSC but lacks an established correlation with neurological recovery, risks producing ROSC in patients who later experience poor neurological outcomes. By focusing on dynamic rhythm change and its association with favorable neurological outcome, our study aligns with the definitive clinical endpoint and provides evidence more directly relevant to decision-making regarding ECPR candidacy in routine clinical practice.

## Data Availability

No datasets were generated or analysed during the current study.

## Abbreviations

CI: Confidence interval
ECPR: Extracorporeal cardiopulmonary resuscitation
OHCA: Out-of-hospital cardiac arrest
OR: Odds ratio
PEA: Pulseless electrical activity
POCUS: Point-of-care ultrasound
ROSC: Return of spontaneous circulation
